# Inhibition of negative feedback for persistent epithelial cell–cell junction contraction by p21-activated kinase 3

**DOI:** 10.1038/s41467-022-31252-0

**Published:** 2022-06-20

**Authors:** Hiroyuki Uechi, Kazuki Fukushima, Ryota Shirasawa, Sayaka Sekine, Erina Kuranaga

**Affiliations:** 1 Laboratory for Histogenetic Dynamics, Graduate School of Life Sciences, Tohoku University, 6-3, Aramaki-aza-aoba, Aoba-ku, Sendai, 980-8578 Japan; 2 Present Address: Max Planck Institute of Molecular Cell Biology and Genetics, 01307 Dresden, Germany

**Keywords:** Cadherins, Morphogenesis, Actin

## Abstract

Actin-mediated mechanical forces are central drivers of cellular dynamics. They generate protrusive and contractile dynamics, the latter of which are induced in concert with myosin II bundled at the site of contraction. These dynamics emerge concomitantly in tissues and even each cell; thus, the tight regulation of such bidirectional forces is important for proper cellular deformation. Here, we show that contractile dynamics can eventually disturb cell–cell junction contraction in the absence of p21-activated kinase 3 (Pak3). Upon Pak3 depletion, contractility induces the formation of abnormal actin protrusions at the shortening junctions, which causes decrease in E-cadherin levels at the adherens junctions and mislocalization of myosin II at the junctions before they enough shorten, compromising completion of junction shortening. Overexpressing E-cadherin restores myosin II distribution closely placed at the junctions and junction contraction. Our results suggest that contractility both induces and perturbs junction contraction and that the attenuation of such perturbations by Pak3 facilitates persistent junction shortening.

## Abstract (web summary)

Actin and myosin operate at cell–cell junctions during junctional shortening. Here the authors show that prolonged actomyosin contractility can compromise junctional shortening, and that Pak3 is required for attenuation of abnormal active protrusive structure and thus keeps junction contraction, appropriate E-cadherin distribution, and junction shortening in Drosophila.

## Introduction

The cell collectives composing animal bodies sculpt tissue architectures through various cellular behaviors such as cell division, deformation, rearrangement, and migration. The cytoskeletal protein actin is the central protein that drives these cellular behaviors by generating mechanical forces^1–7^. While actin generates protrusive forces by forming branched and bundled structures, it also supplies contractile forces by forming bundled structures and loosely organized networks in concert with non-muscle myosin II. Such bidirectional force generation by actin can coexist in each cell and even at the same position within cells. During single-cell migration, protrusive actin dynamics extend cells at the leading edge, while actomyosin (the actin and myosin II complex) contraction causes retraction at the rear of the cell along the migrating direction^8,9^. A recent study of *Drosophila* eye development demonstrated that pulsatile extension by protrusive branched actin networks and counterbalancing actomyosin contractility-mediated shortening of each cell–cell contact control cellular shape^10^. Thus, the tight regulation of actin dynamics is important for proper force induction and the resultant cellular dynamics.

**Fig. 1:**
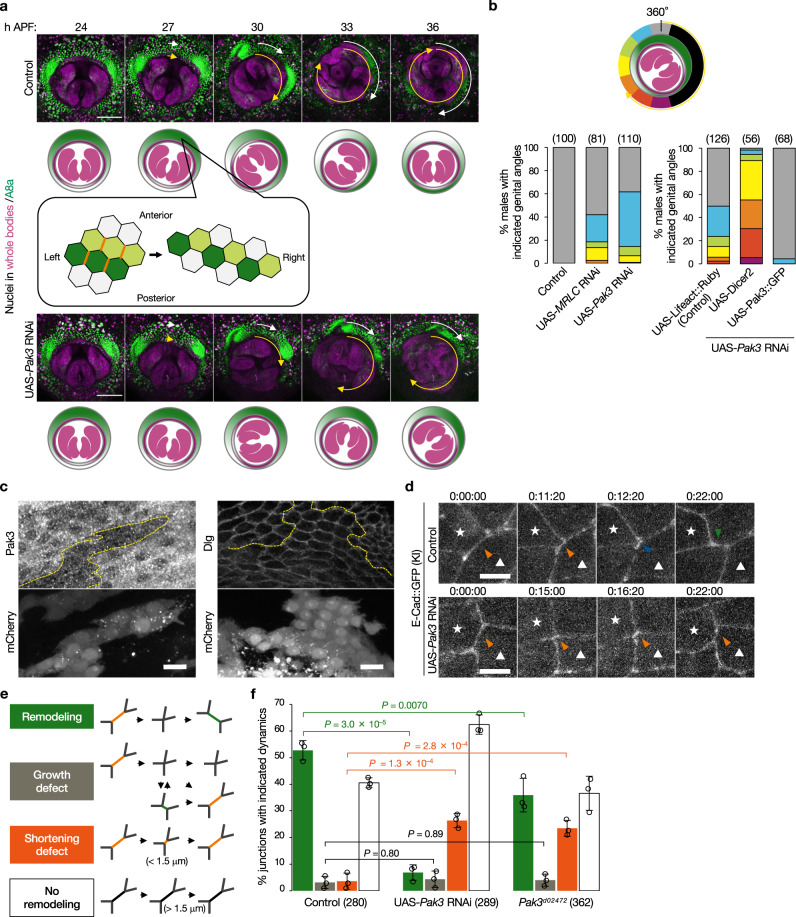
Pak3 is required for cell–cell junction shortening during epithelial junction remodeling. **a** Top, representative time-lapse images of male genitalia observed from the posterior end during rotation from >3 independent experiments. The ventral side is located at the top. Nuclei in the A8a cells and the whole body are depicted in green and purple, respectively. Yellow and white arrows indicate the movements of genitalia and the A8a epithelia, respectively. Scale bar, 100 μm. Bottom, a schematic illustration showing the angles of genitalia with the A8p epithelia (both purple) and the A8a epithelia (green), and a schematic illustration of cell intercalation in the A8a epithelia. Orange lines indicate shortening junctions. **b** Top, a schematic illustration of the categorization of genitalia angles. Yellow arrow indicates genitalia movement. Gray indicates the 360° rotation of genitalia (normal orientation). Bottom, percentages of male adult flies with the indicated genitalia angles are shown. Parentheses, the number of males examined. **c** Representative images of A8a epithelia at 28 h APF immunostained for Pak3 and Dlg from two independent experiments. Pak3 RNAi cells are indicated with mCherry signals. Scale bar, 10 μm. Broken lines indicate the edges of Pak3 RNAi clones. **d** Representative time-lapse images of E-Cad::GFP at remodeling junctions from >3 independent experiments. Stars and triangles indicate cells forming the shortening junctions. Orange, blue, and green arrowheads indicate shortening junctions, four-way vertices, and growing junctions, respectively. Scale bar, 5 μm. **e** A schematic illustration of the categorization of the junction dynamics. **f** Mean ± S.D. of the percentages of junctions with the categorized dynamics. Bar colors correspond to **e**. Parentheses, the number of examined junctions from *n* = 3 biologically independent pupae per genotype. *P-*values by two-tailed Dunnett’s test. Genotypes: **a** +*/Y;His2Av::mRFP/+;AbdB-Gal4, UAS-H2B::ECFP/*+ (Control) and +*/Y;His2Av::mRFP/+;AbdB-Gal4, UAS-H2B::ECFP/UAS-Pak3 RNAi*; **b** (bottom left) *+/Y;;AbdB-Gal4/+* (Control), *+/Y;;AbdB-Gal4/UAS-MRLC RNAi*, and +*/Y;;AbdB-Gal4/UAS-Pak3 RNAi*; (bottom right) *+/Y;UAS-Lifeact::Ruby/+;AbdB-Gal4/UAS-Pak3 RNAi* (Control), *+/Y;UAS-Dicer2/+;AbdB-Gal4/UAS-Pak3 RNAi*, and +*/Y;;AbdB-Gal4, UAS-Pak3::GFP/UAS-Pak3 RNAi*; **c** *hs-flp/Y;E-Cad::GFP (KI);Act* > *CD2* > *Gal4, UAS-mCD8::mCherry/UAS-Pak3 RNAi*; **d**, **f** +*/Y;E-Cad::GFP (KI);AbdB-Gal4, UAS-H2B::ECFP/*+ (Control), *+/Y;E-Cad::GFP (KI);AbdB-Gal4, UAS-H2B::ECFP/UAS-Pak3 RNAi*, and +*/Y;E-Cad::GFP;Pak3*^*d02472*^. Source data are provided as a Source data file.

Epithelial cell intercalation is one of the multicellular dynamics driven by the contractile forces of actomyosin and contributes to directional tissue extension and movement^11–13^. This process consists of the directional exchange of cellular positions within cell collectives, which is driven by cell–cell junction remodeling: i.e., shortening of cell–cell junctions and subsequent growth of new ones in new directions (Fig. 1a)^14^. During shortening, actin and myosin II are highly enriched at the adherens junctions (AJs) of cell–cell junctions to form contractile actomyosin bundles and then shorten the junctions^1,11,15–19^. The mechanisms inducing contractile forces via actomyosin are well studied; however, it is still unknown whether actomyosin-mediated contractions are negatively regulating cell–cell junction shortening, and if so, how the shortening is sustained.

We have previously shown that epithelial intercalation is observed during morphogenesis of *Drosophila* male genitalia^20,21^. During metamorphosis, male genitalia located at the posterior end of the body undergo 360° dextral rotation around the anterior–posterior axis (Fig. 1a)^21,22^. This rotation is observed from 24 to 36 h after puparium formation (APF), and is composed of two movements of epithelial cells: the initial 180° dextral movement of the genitalia along with the surrounding epithelia, which is called the posterior compartment of the A8 segment (A8p), and the subsequent 180° dextral movement of the anterior compartment of the A8 segment (A8a), the latter of which starts around 2 h after initiation of the A8p movement (26 h APF)^20–22^. During rotation, the A8a cells frequently induce left–right polarized junction remodeling in relation to the anterior–posterior axis in the confined space between the A8p epithelia and the A7 segment, which results in unidirectional epithelial cell movement (Fig. 1a)^20,23^. Consistent with other tissues, the accumulation of actomyosin at the AJs of cell–cell junctions induces junction contraction in this model. Down-regulation of contractile activity, such as via RNA interference (RNAi) of myosin II regulatory light chain (MRLC), compromises the remodeling and hence results in insufficient A8a cell movement, leading to incomplete rotation of the genitalia^20^.

We herein examined the actin dynamics during cell intercalation in the A8a epithelia and show that, upon junction contraction, the actin dynamics at the cell–cell junctions are compromised in p21-activated kinase 3 (Pak3) mutant flies. Pak proteins belong to a family of serine/threonine kinases that are involved in various cellular functions via their regulation of cytoskeletal dynamics^24–27^. *Drosophila* Pak3 is reported to have roles in multicellular dynamics in the context of fly development, including embryonic dorsal closure, wound healing of the larval epidermis, larval myoblast cell–cell fusion, and border cell migration in the oocyte^28–31^. In the A8a epithelia, Pak3 depletion causes aberrant actin protrusions that disturb the distribution of E-cadherin and myosin II at the junctions, and thus eventually disrupts the junction contraction. These findings suggest that Pak3 blocks the negative feedback of contractility and ensures persistent junction contraction and rearrangement of epithelial cells.

## Results

### Pak3 is required for A8a cell movement during the genitalia rotation

To understand the molecular basis underlying epithelial cell–cell junction remodeling, we searched for genes involved in the movement of A8a cells. In the control flies, time-lapse images showed that male genitalia underwent 180° rotation with respect to the A8a epithelia, which also showed 180° movement, resulting in 360° full rotation during metamorphosis (Fig. 1a). After eclosion, the orientation of the genitalia was normal in all control male flies (Fig. 1b). We found that RNAi targeting Pak3 in the A8a epithelia, induced by the UAS/Gal4 system with the *AbdB-Gal4* driver, caused insufficient movement of the A8a epithelia during rotation (Fig. 1a)^32,33^. In contrast, the genitalia still underwent 180° movement independently with the A8a epithelia, as observed in the control flies, indicating that only the A8a movement was impaired (Fig. 1a). The depletion of Pak3 eventually induced the misorientation of genitalia in ~60% of adult male flies, which was similar to the frequency reported in association with MRLC depletion (Fig. 1b)^20^. Misorientation was also observed with another Pak3 RNAi strain (Supplementary Fig. 1). The co-expression of Dicer2, which augments RNAi efficiency^34^, with Pak3 RNAi increased the frequency of misorientation to >90%, which was associated with a decline in rotation angle (Fig. 1b). Expressing Dicer2 alone did not induce the orientation defect (Supplementary Fig. 1). The overexpression of Pak3 tagged with green fluorescent protein (Pak3::GFP) restored the normal orientation, while the co-expression of a red fluorescent protein-tagged Lifeact (Lifeact::Ruby), which labels F-actin^35^ and is used as a control in this experiment, had a marginal effect on the orientation defect (Fig. 1b). These results indicate that Pak3 is involved in dynamics of the A8a epithelia.

Immunostaining of fixed epithelia revealed that Pak3 was expressed in the A8a cells, as indicated by its decreased signals in Pak3 RNAi cells that were clonally introduced into wild-type tissues (Fig. 1c). Discs large (Dlg) is a lateral membrane-associated protein and a component of the septate junction, which corresponds to the tight junction in mammalian cells^36^. Signals of Dlg at cell–cell boundaries were indistinguishable between wild-type cells and Pak3 RNAi clones, suggesting that Pak3 RNAi does not compromise the integrity of epithelial cells (Fig. 1c).

### Pak3 is required for cell–cell junction shortening

To examine the role of Pak3 in A8a cell movement, we monitored the dynamics of the cell–cell junctions using a knock-in (KI) strain of a central AJ component E-cadherin tagged with GFP (E-Cad::GFP)^37,38^. In the control epithelia, we observed that cell–cell junctions undergo shortening, form four-way vertices, and subsequently grow in other directions, consistent with previous reports (Fig. 1d; Supplementary Movie 1)^20,39^. In the Pak3-depleted A8a cells, junctions frequently failed to complete shortening: they did not shorten sufficiently to form four-way vertices and instead went back to the original direction (Fig. 1d; Supplementary Movie 2). To quantify these defective junction dynamics, we categorized the junctions in A8a cells according to their dynamics as follows: junctions that completed shortening and grew in other directions (remodeling), junctions that completed shortening but failed to grow in other directions after forming four-way vertices (growth defect), junctions that shortened sufficiently to reach 1.5 μm in length but failed to form four-way vertices (shortening defect), and junctions that did not shorten to <1.5 μm in length (no remodeling) (Fig. 1e). Then, the populations of junctions in each category were examined. In the control cells, ~55% of junctions underwent remodeling, and few junctions showed defects during a 4-h period following the initiation of A8a cell movement (from 26 to 30 h APF, Fig. 1f). By contrast, in Pak3 RNAi cells, the frequency of junction remodeling was decreased to <10%, and instead the number of shortening junctions that failed to form four-way vertices (categorized as a shortening defect) was significantly increased (Fig. 1f). Similar propensities were observed in a Pak3 hypomorphic mutant (*Pak3*^*d02472*^)^40^: flies with this mutation showed a modest decline in the frequency of junction remodeling, whereas the shortening defect occurred as frequently as in Pak3 RNAi cells (Fig. 1f). Consistent with this modest junction remodeling defect, *Pak3*^*d02472*^ flies did not show misorientation of male genitalia (*n* > 100) in contrast to the Pak3 RNAi flies. This is probably due to the difference in depletion efficiency of Pak3: while Pak3 RNAi expression in the whole body with the *Actin-Gal4* driver induced complete lethality at the pupal stage (*n* > 100), a previous report showed that ~10% of *Pak3*^*d02472*^ homozygous adults emerged^40^. Also, we speculate that there is a critical remodeling frequency (threshold) that determines whether the A8a epithelia fully move to 180° or not, although it is not characterized yet. Although both Pak3 RNAi and hypomorphic mutation decreased the frequency of the remodeling, that of the growth step was not altered in these Pak3 mutants (Fig. 1f). Taken together, these results suggest that Pak3 is required for junction shortening during cell intercalation.

### Pak3 suppresses aberrant actin dynamics upon junction contraction

**Fig. 2:**
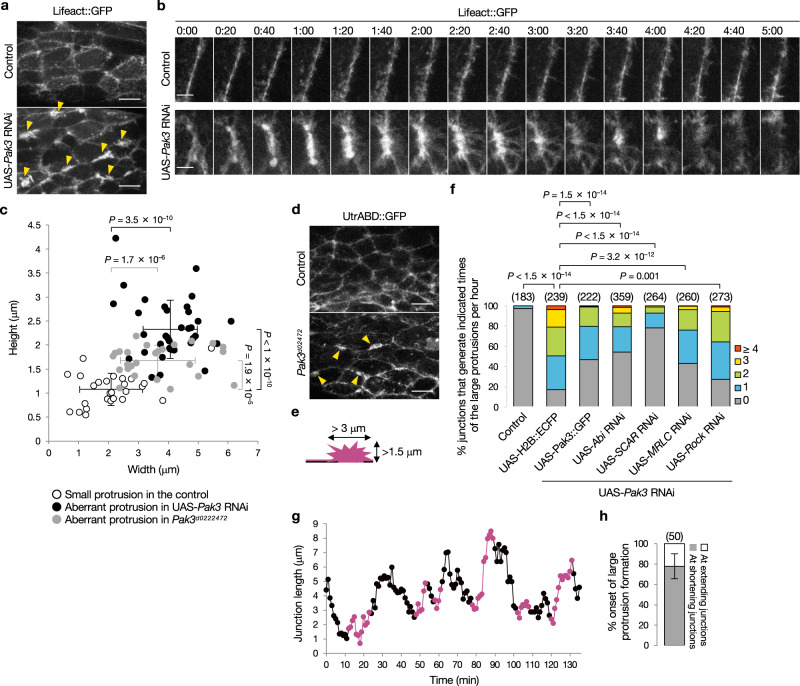
Pak3 depletion induces aberrant actin dynamics. **a** Representative images of actin labeled with Lifeact::GFP in the A8a cells from >3 independent experiments. Arrowheads indicate some aberrant actin protrusions. Scale bar, 10 μm. **b** Magnified time-lapse images of Lifeact::GFP at cell–cell junctions, representative of >3 independent experiments. Scale bar, 3 μm. **c** Mean ± S.D. of the height and width of small actin protrusions in the control cells and aberrant protrusions in the Pak3-depleted cells. *n* = 26 (control and Pak3 RNAi) or 36 (*Pak*^*d02472*^) protrusions from 5 (Control and Pak3 RNAi) or 2 (*Pak3*^*d02472*^) biologically independent pupae per genotype. *P-*values by two-tailed Dunnett’s test. **d** Representative images of actin labeled with UtrABD::GFP in the A8a cells from two independent experiments. Arrowheads indicate some aberrant actin protrusions. Scale bar, 10 μm. **e** A schematic illustration of the large actin protrusions (magenta) at a junction (black). **f** Percentages of junctions generating the large actin protrusions for the indicated number of times per hour are shown. Parentheses, the number of examined junctions from 4 to 5 pupae per genotype. *P-*values by two-tailed Mann–Whitney *U*-test. **g** Graph showing the length of a representative junction that repeats shortening and re-extension in Pak3 RNAi cells. The timings when the large actin protrusions emerge are shown in magenta. **h** Mean ± S.D. of the timing of the onset of the large actin protrusion formation. Parenthesis indicates the number of protrusions from 15 junctions of 3 pupae. Genotypes: **a**–**c** +*/Y;UAS-Lifeact::GFP/*+*;AbdB-Gal4/+* (Control) and +*/Y;UAS-Lifeact::GFP/*+*;AbdB-Gal4/UAS-Pak3 RNAi*; **d** +*/Y;sqh-UtrABD::GFP/*+ (Control) and +*/Y;sqh-UtrABD::GFP/*+*;Pak3*^*d02472*^; **f** +*/Y;UAS-Lifeact::GFP/*+*;AbdB-Gal4/+* (Control), *+/Y;UAS-Lifeact::GFP/*+*;AbdB-Gal4, UAS-H2B::ECFP/UAS-Pak3 RNAi*, *+/Y;UAS-Lifeact::GFP/*+*;AbdB-Gal4, UAS-Pak3::GFP/UAS-Pak3 RNAi*, *+/Y;UAS-Lifeact::GFP/UAS-Abi RNAi;AbdB-Gal4/UAS-Pak3 RNAi*, *+/Y;UAS-Lifeact::GFP/UAS-SCAR RNAi;AbdB-Gal4/UAS-Pak3 RNAi*, *+/Y;UAS-Lifeact::GFP/UAS-MRLC RNAi;AbdB-Gal4/UAS-Pak3 RNAi*, and +*/Y;UAS-Lifeact::GFP/UAS-Rock RNAi;AbdB-Gal4/UAS-Pak3 RNAi*; **g**, **h** +*/Y;UAS-Lifeact::GFP/*+*;AbdB-Gal4/UAS-Pak3 RNAi*. Source data are provided as a Source data file.

Junction shortening is driven by the contractile forces of actomyosin accumulating at the AJs of junctions, and Pak protein families are known to regulate actin dynamics^5,24,25,41^. These findings and our observations led us to speculate that Pak3 has roles in actin dynamics during junction shortening in the A8a epithelia. To test this possibility, we performed time-lapse imaging of GFP-tagged Lifeact (Lifeact::GFP)^35^. In the control A8a cells, actin was distributed predominantly at cell–cell junctions (Fig. 2a; Supplementary Movie 3). Magnified images showed that, whereas the majority of Lifeact::GFP signals were localized along junctions, actin frequently generated small protrusions arising from the junctions (Fig. 2b). The maximum size of these small protrusions was 1.1 ± 0.34 μm in height (perpendicular to the junctions) and 2.1 ± 1.1 μm in width (parallel to the junctions) (Fig. 2c). These observations suggest that actin not only merely forms into bundled structures along cell–cell junctions but also generates protrusive structures in the A8a cells undergoing junction remodeling. In Pak3 RNAi cells, actin similarly localized to the cell–cell junctions and formed small protrusions. In addition, Pak3-depleted cells had prominent protrusive structures that were larger than the small protrusions in both height and width (Fig. 2a–c; Supplementary Movie 4). Such aberrant actin-positive structures were also observed in the other Pak3 RNAi strains as well as in *Pak3*^*d02472*^ flies in which actin was labeled with GFP-tagged actin-binding domain of utrophin (UtrABD::GFP) (Fig. 2c, d and Supplementary Fig. 2a)^42,43^. To semi-quantify these actin dynamics in Pak3 RNAi cells, we defined a “large” actin protrusion as a Lifeact-positive cluster at junctions that was >1.5 μm in height and >3 μm in width in a planar section; both lengths approximately exceeded the mean + 1 standard deviation (S.D.) of each length of the small actin protrusions in the control cells, respectively (Fig. 2c, e). We then examined the frequency of the appearance of the large protrusions at each junction (Fig. 2f). While >97% of cell–cell junctions did not generate such large protrusions in the control cells, they emerged at >80% and ~30% of junctions at least once per hour in Pak3 RNAi and *Pak3*^*d02472*^ cells, respectively (Fig. 2f and Supplementary Fig. 2b). Since the phenotypes at junctions, i.e., defect of junction remodeling and frequent emergence of the large protrusions, were more prominent in Pak3 RNAi than *Pak3*^*d02472*^, associated with genitalia rotation defect, we hereafter focused on the Pak3 RNAi strain.

Overexpressing Pak3 partially alleviated the formation of the large protrusions: although these structures were still observed in ~50% of junctions, they were absent from >40% of junctions, and only a few junctions formed the large protrusions more than three times per hour (Fig. 2f). Since the large actin protrusions were characteristic of Pak3-depleted cells, we subsequently focused on these aberrant actin dynamics.

Branched actin networks induce the formation of protrusive actin structures and are generated by the Arp2/3 complex, which is stimulated by the WAVE regulatory complex (WRC)^1,3^. To determine whether this pathway is involved in the formation of the large protrusions, we used RNAis targeting Abi or SCAR, components of the WRC, in Pak3-depleted cells^44,45^. The depletion of Abi suppressed the generation of large actin protrusions in Pak3 RNAi cells, while depletion of Abi alone did not induce large protrusions (Fig. 2f and Supplementary Fig. 2c). The depletion of SCAR also suppressed the emergence of large protrusions (Fig. 2f). These results suggest that the aberrant actin protrusions in Pak3-depleted cells are in part composed of branched actin networks.

To further characterize the aberrant actin dynamics, we explored the correlation between emergence of the large protrusions and junction dynamics. In Pak3 RNAi cells, junctions failed to undergo remodeling (Fig. 1f) but instead underwent repeated shrinkage and extension (Fig. 2g). We found that the generation of the large protrusions was initiated frequently when the junctions were shortening rather than when they were extending (Fig. 2g). Approximately 80% of the large actin protrusions emerged at shortening junctions (Fig. 2h). On the other hand, there was no obvious correlation between emergence of small protrusions and junction dynamics (Supplementary Movies 3, 4). These observations raise the possibility that junction contraction sensitizes cells to the formation of aberrant protrusions in the absence of Pak3. To evaluate this possibility, we depleted MRLC or Rock, an upstream activator of myosin II^1,5^. The depletion of these factors did not increase frequency at which the large protrusions were induced, and suppressed their emergence in Pak3 RNAi cells (Fig. 2f and Supplementary Fig. 2c). Collectively, these results suggest that Pak3 suppresses the formation of branched network-containing aberrant actin protrusions at cell–cell junctions upon the contraction of the junctions.

### Pak3 keeps myosin II cables close to cell–cell junctions

**Fig. 3:**
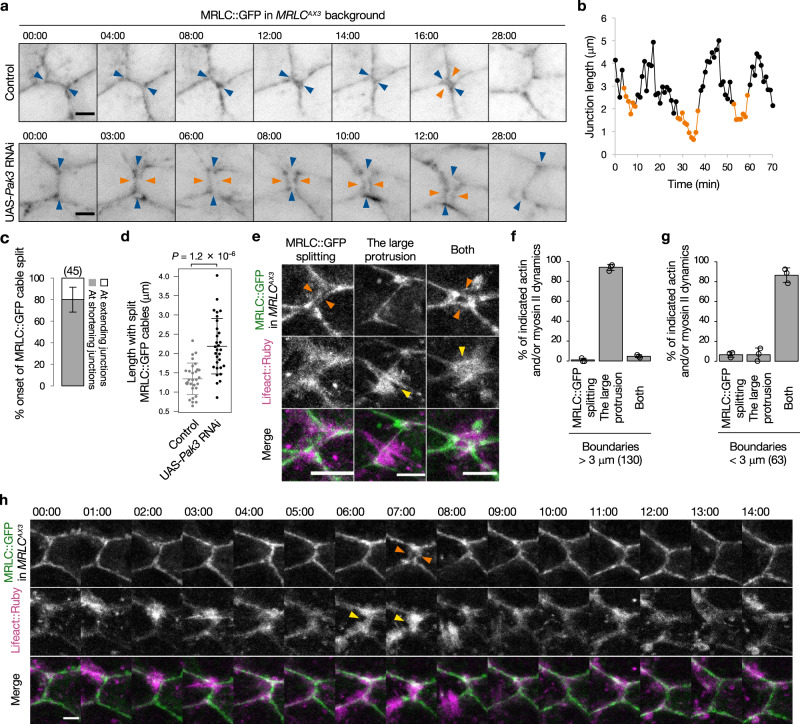
Pak3 depletion causes the mislocalization of myosin II cables. **a** Representative time-lapse images of MRLC::GFP at the shortening junctions from >3 independent experiments. Orange and blue arrowheads indicate splitting myosin II cables and the positions of the edges of the junctions, respectively. Scale bar, 3 μm. **b** Graph showing the length of a junction, and the timings at which the two distinct MRLC::GFP cables split along the junctions are shown in orange. **c** Mean ± S.D. of timing of the onset of MRLC::GFP cable splitting along the junctions. Parentheses, the number of the MRLC::GFP cable splitting from 21 junctions of 3 pupae. **d**, Mean ± S.D. of the lengths of junctions when the MRCL::GFP cables initiated splitting; *n* = 30 junctions from 3 (Control) and 4 (Pak3 RNAi) biologically independent pupae were examined. *P*-value by unpaired *t*-test. **e** Representative images of cell–cell junctions with split MRCL::GFP cables (orange arrowheads), the large actin protrusions (yellow arrowheads), and both in Pak3 RNAi cells (from the left). Scale bar, 5 μm. **f**, **g** Mean ± S.D. of the percentages of the dynamics categorized in (**e**) at cell–c**e**ll junctions larger than (**f**) and smaller than (**g**) 3 μm. Parentheses, the number of examined junctions from *n* = 3 biologically independent pupae. **h** Representative time-lapse images of MRLC::GFP and Lifeact::Ruby at the cell–cell junction in Pak3 RNAi cells. Orange and yellow arrowheads indicate split MRLC::GFP cables and the large actin protrusions, respectively. Scale bar, 3 μm. Genotypes: **a**, **d** *MRLC*^*AX3*^*/Y;MRLC-MRLC::GFP;AbdB-Gal4, UAS-H2B::ECFP/*+ (Control) and *MRLC*^*AX3*^*/Y;MRLC-MRLC::GFP;AbdB-Gal4, UAS-H2B::ECFP/UAS-Pak3 RNAi*; **b**, **c** *MRLC*^*AX3*^*/Y;MRLC-MRLC::GFP;AbdB-Gal4, UAS-H2B::ECFP/UAS-Pak3 RNAi*; **e**–**h** *MRLC*^*AX3*^*/Y;MRLC-MRLC::GFP/UAS-Lifeact::Ruby;AbdB-Gal4, UAS-H2B::ECFP/UAS-Pak3 RNAi*. Source data are provided as a Source data file.

In Pak3 RNAi cells, after the emergence of large actin protrusions, the junctions failed to continue shortening and instead re-extended (Fig. 2g), implying that the large actin protrusions induced upon junction shortening in turn perturb contraction. To understand how junction contraction is compromised, we examined the dynamics of myosin II using GFP-tagged MRLC (MRLC::GFP) in an endogenous MRLC-depleted background (*MRLC*^*AX3*^), with which we avoided unnecessary competition between tagged and endogenous MRLC proteins^46^. Time-lapse imaging of MRLC::GFP showed that the majority of MRLC::GFP signals were observed as a single cable at cell–cell junctions (Fig. 3a). In Pak3 RNAi cells, we observed MRLC::GFP signals split into two distinct cables along junctions (Fig. 3a, orange arrowheads). The splitting was predominantly initiated at shortening junctions rather than at extending junctions, and eventually the two cables were completely split along the junctions (Fig. 3a–c). In the control cells, such MRLC::GFP cable splitting was only observed when the length of shortening junctions was sufficiently decreased (Fig. 3a, orange arrowheads). Measurement of the length of junctions when the MRLC::GFP cables initiate splitting at the junction showed that Pak3 RNAi increased this length from 1.3 ± 0.41 to 2.2 ± 0.72 μm, showing that the MRLC::GFP cables split earlier during junction shortening in Pak3 RNAi cells (Fig. 3d). Since E-Cad::GFP signals did not form into such split cables along the junctions (Fig. 1d and Supplementary Fig. 3), splitting of the MRLC::GFP cables is not likely due to detachment of the cells. Collectively, these observations suggest that localization of myosin II is compromised at the shortening cell–cell junctions in Pak3 RNAi cells.

**Fig. 4:**
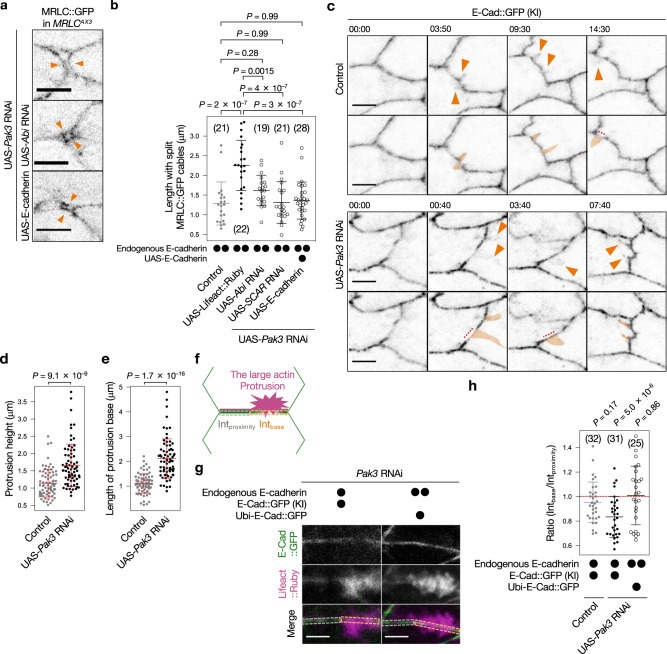
Decrease in E-cadherin levels at junctions mediates the mislocalization of myosin II cables. **a** Representative images of split MRLC::GFP cables (orange arrowheads) at junctions. Scale bar, 5 μm. **b** Mean ± S.D. of the lengths of junctions when the MRLC::GFP cables initiated splitting. Dots indicate the copy number of each E-cadherin allele. Parentheses, the number (*n*) of examined junctions from 3–6 biologically independent pupae per genotype. *P*-value by two-tailed Tukey’s test. **c** Representative magnified time-lapse images (duplicated) of E-Cad::GFP at junctions from >3 independent experiments. E-Cad::GFP-positive protrusions are indicated by arrowheads (top) or masked (bottom), respectively. Broken lines indicate the local decrease in E-Cad::GFP levels at the bases of the protrusions. Scale bar, 3 μm. **d**, **e** The mean ± S.D. of the height and width (length of the base) of E-Cad::GFP-positive protrusions. *n* = 77 (Co*n*trol) or 74 (Pak3 RNAi) protrusions from 5 (Control) and 7 (Pak3 RNAi) biologically independent pupae (**c**–**e**). *P*-value by unpaired *t*-test. **f** A schematic illustration of the junctional regions (boxed with broken lines) for measuring the mean E-Cad::GFP levels at the base of small actin protrusions in the control cells or the large protrusions in Pak3 RNAi cells (orange), and its proximity (gray). **g** Images of junctions with E-Cad::GFP and Lifeact::Ruby in Pak3 RNAi cells. Dots indicate the copy number of each E-cadherin allele. Broken boxes indicate quantified areas; each color corresponds with the colors in (**f**). Scale bar, 3 μm. **h** Mean ± S.D. of the ratio of Int_base_ and Int_proximity_ per junction. Parentheses, the number (*n*) of small (control) or the large (Pak3 RNAi) actin protrusions examined from 4 (the control and Pak3 RNAi) and 3 (Pak3 RNAi with Ubi-E-Cad::GFP) biologically independent pupae. Dots indicate the copy number of each E-cadherin allele. *P*-value by one-sample *t*-test with a test value of 1. Genotypes: **a**, **b** *MRLC*^*AX3*^*/Y;MRLC-MRLC::GFP/*+*;AbdB-Gal4, UAS-H2B::ECFP/*+ (Control), *MRLC*^*AX3*^*/Y;MRLC-MRLC::GFP/UAS-Lifeact::Ruby;AbdB-Gal4, UAS-H2B::ECFP/UAS-Pak3 RNAi*, *MRLC*^*AX3*^*/Y;MRLC-MRLC::GFP/UAS-Abi RNAi;AbdB-Gal4, UAS-H2B::ECFP/UAS-Pak3 RNAi*, *MRLC*^*AX3*^*/Y;MRLC-MRLC::GFP/UAS-SCAR RNAi;AbdB-Gal4, UAS-H2B::ECFP/UAS-Pak3 RNAi*, and *MRLC*^*AX3*^*/Y;MRLC-MRLC::GFP/UAS-E-cadherin;AbdB-Gal4, UAS-H2B::ECFP/UAS-Pak3 RNAi*; **c**–**e** +*/Y;E-Cad::GFP;AbdB-Gal4, UAS-H2B::ECFP/*+ (Control) and +*/Y;E-Cad::GFP (KI);AbdB-Gal4, UAS-H2B::ECFP/UAS-Pak3 RNAi*; **g**, **h** +*/Y;E-Cad::GFP (KI)/UAS-Lifeact::Ruby;AbdB-Gal4/+* (Control), *+/Y;E-Cad::GFP (KI)/UAS-Lifeact::Ruby;AbdB-Gal4/UAS-Pak3 RNAi*, and +*/Y;Ubi-E-Cad::GFP/UAS-Lifeact::Ruby;AbdB-Gal4/UAS-Pak3 RNAi*. Source data are provided as a Source data file.

The initiation of both the large actin protrusions and split MRLC::GFP cables was predominantly observed at shortening junctions (Figs. 2h and 3c). To examine their interplay, we labeled myosin II and actin simultaneously using MRLC::GFP and Lifeact::Ruby^35^ in the *MRLC*^*AX3*^ background of Pak3 RNAi cells, and their aberrant dynamics were categorized into the following three groups: only splitting of MRLC::GFP signals, only generation of the large actin protrusions, and both (Fig. 3e). At junctions of >3 μm in length, which roughly exceeded the mean + 1 S.D. of the length at which the MRLC::GFP cables were split in Pak3-depleted cells, large actin protrusions were generated in the absence of MRLC::GFP cable splitting (Fig. 3d, f). In contrast, at junctions of <3 μm in length (or shortening junctions), most of the splitting cables were observed concomitantly with the large actin protrusions (Fig. 3g). Furthermore, the emergence of large actin protrusions preceded splitting of the MRLC::GFP cables (Fig. 3h). These observations raise the possibility that aberrant actin dynamics upon Pak3 depletion triggers the mislocalization of myosin II cables at shortening junctions. To test this possibility, we suppressed the formation of the large actin protrusions by additional RNAis of Abi and SCAR (Fig. 2f). These manipulations reduced the junction length at which the MRLC::GFP cables initiated splitting (Fig. 4a, b). Taken together, these results suggest that Pak3 keeps myosin II cables close to the cell–cell junctions by inhibiting the formation of aberrant actin protrusions.

### Junctional E-cadherin levels mediate the myosin II mislocalization

To clarify how the aberrant actin dynamics and consequent mislocalization of myosin II cables at shortening junctions are connected, we again explored the distribution of E-cadherin, since it is a central component of the cadherin–catenin core complex that acts as a scaffold for actomyosin at the AJs^38,47^. Magnified time-lapse images of E-Cad::GFP (KI) in the control cells revealed that while the majority of E-Cad::GFP signals were localized along cell–cell junctions, they frequently formed small protrusions arising from the junctions (Fig. 4c; Supplementary Movie 5). Co-imaging of E-Cad::GFP (KI) and Lifeact::Ruby showed that ~75% of small protrusions were positive for both signals, ~20% were positive for only Lifeact::Ruby, and <5% were positive for only E-Cad::GFP-positive, suggesting that E-cadherin protrusions are derived from actin dynamics (Supplementary Fig. 4a, b). The junctions in Pak3-depleted cells also generated E-Cad::GFP-positive protrusions; they were larger in height and width (the length of the base of protrusions along the junctions) (Fig. 4c–e; Supplementary Movie 6). We also observed that the E-Cad::GFP levels were sometimes locally decreased at the base of these protrusions on the junctions (Fig. 4c, broken line). To assess whether actin protrusions reduced the local levels of junctional E-cadherin, we compared the junctional E-Cad::GFP levels at the base of small actin protrusions in the control cells or the large actin protrusions in Pak3 RNAi cells (Int_base_) with those at the region in proximity to the protrusions (Int_proximity_) along each junction (Fig. 4f). The Int_base_ and Int_proximity_ were comparable in the control cells, as the Int_base_/Int_proximity_ ratio was ~1 (Fig. 4h). In contrast, the ratio was significantly below 1 in Pak3 RNAi cells (Fig. 4g, h). This suggests that local aberrant protrusions rather than small protrusions decrease the junctional E-cadherin levels.

We then asked whether the decrease in junctional E-cadherin levels is a cause of the mislocalization of myosin II. We first depleted E-cadherin and another AJ component, β-catenin. These manipulations frequently induced MRLC::GFP cable splitting (Supplementary Fig. 4c). Such myosin II cable splitting in association with the decrease in E-cadherin levels is also observed during the elimination of apoptotic cells from epithelia^48^. Next, we increased E-cadherin levels by (i) adding one more copy of full-length E-cadherin tagged with GFP using a ubiquitin promotor-linked strain (Ubi-E-Cad::GFP)^49^, which increased total E-cadherin mRNA levels (Supplementary Fig. 4d), or (ii) overexpression using UAS-E-cadherin (non-tagged full-length E-cadherin) and UAS-E-Cad::GFP (GFP-tagged full-length E-cadherin)^50^. The introduction of Ubi-E-Cad::GFP in Pak3 RNAi cells increased the Int_base_/Int_proximity_ ratio toward ~1, suggesting that E-cadherin upregulation mitigates the local reduction in junctional E-cadherin levels upon the formation of aberrant actin dynamics (Fig. 4f–h). The overexpression of non-tagged E-cadherin in Pak3 RNAi cells decreased the junction length at which the junction initiated splitting of MRLC::GFP cables (Fig. 4a, b). In contrast, the introduction of neither Ubi-E-Cad::GFP nor UAS-E-Cad::GFP attenuated the generation of large actin protrusions, indicating that the local decrease in E-cadherin levels is located downstream from the formation of aberrant actin protrusions (Supplementary Fig. 4e, f). Taken together, these results suggest that the decrease in E-cadherin levels at cell–cell junctions induced by aberrant actin dynamics causes the inability of Pak3-depleted cells to keep myosin II close to the junctions.

### The overexpression of E-cadherin restores multicellular movement

**Fig. 5:**
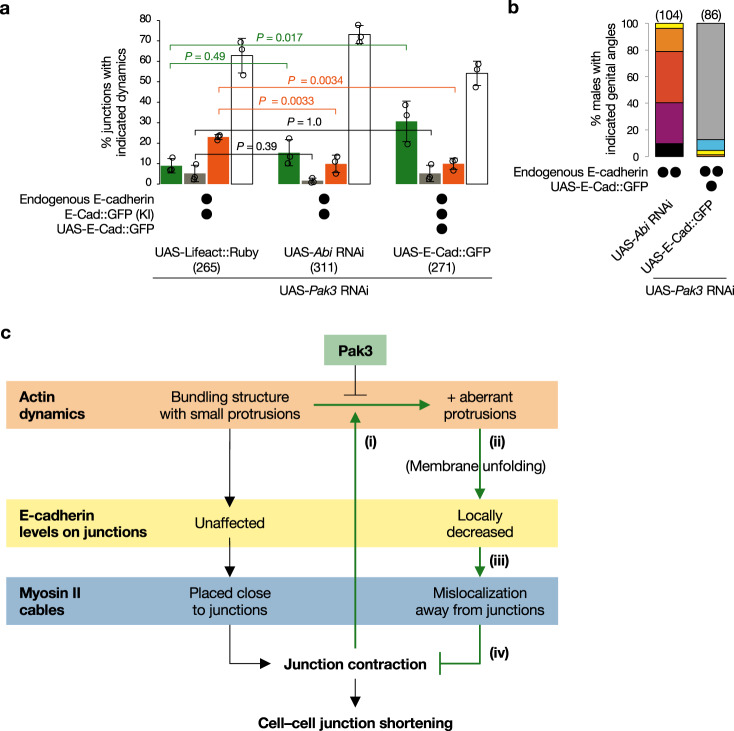
E-cadherin overexpression rescues tissue dynamics. **a** Mean ± S.D. of the percentages of junctions with the categorized dynamics. Bar colors correspond to Fig. 1e. Dots indicate the copy number of each E-cadherin allele. Parentheses, the number of examined junctions from *n* = 3 biologically independent pupae per genotype. *P-*values by two-tailed Dunnett’s test. **b** Percentages of male adult flies with genitalia angles indicated in Fig. 1b are shown. Dots indicate the copy number of each E-cadherin allele. Parentheses, the number of males examined. **c** A schematic illustration of Pak3-dependent junction contraction. (i)–(iv) indicate the negative feedback mechanism (green arrows, described in the “Discussion” section). Genotypes: **a** +*/Y;E-Cad::GFP (KI)/UAS-Lifeact::Ruby;AbdB-Gal4, UAS-H2B::ECFP/UAS-Pak3 RNAi*, *+/Y;E-Cad::GFP (KI)/UAS-Abi RNAi;AbdB-Gal4, UAS-H2B::ECFP/UAS-Pak3 RNAi*, and +*/Y;E-Cad::GFP (KI)/UAS-E-Cad::GFP;AbdB-Gal4, UAS-H2B::ECFP/UAS-Pak3 RNAi*; **b** +*/Y;UAS-Abi RNAi/+;AbdB-Gal4/UAS-Pak3 RNAi* and +*/Y;UAS-E-Cad::GFP/*+*;AbdB-Gal4/UAS-Pak3 RNAi*. Source data are provided as a Source data file.

The depletion of Abi and the overexpression of E-cadherin suppressed the mislocalization of myosin II (Fig. 4a, b). We finally explored whether these manipulations also restored the tissue dynamics in Pak3 RNAi flies. Abi RNAi decreased the frequency of the shortening defect and marginally increased the junction-remodeling frequency in Pak3 RNAi cells, although the latter increase was not statistically significant (Fig. 5a). However, Abi RNAi did not mitigate the genitalia orientation defect in Pak3 RNAi adult males; rather, it exacerbated the defect (Figs. 1b and 5b). This result may reflect non-significant rescue of the junction-remodeling frequency, and suggests that branched actin structures have roles in the A8a movement in mechanisms other than junction remodeling.

On the other hand, the overexpression of E-Cad::GFP increased the frequency of junction-remodeling and decreased the frequency of the shortening defect in Pak3 RNAi cells (Fig. 5a). In addition, this manipulation decreased the number of adult males with the genitalia showing an abnormal orientation (Figs. 1b and 5b). These results suggest the E-cadherin-mediated close localization of myosin II cables to junctions is required for the completion of junction shortening, leading to proper cell intercalation and epithelial cell movement.

## Discussion

During cell–cell junction shortening, actin and myosin II accumulate at junctions, and the resultant actomyosin bundles generate contractile forces^5,51^. In contrast, previous reports demonstrated that myosin II contractility potentiates actin unbundling and depolymerization in vitro and in vivo^52,53^, which could compromise contraction. It has also been recently shown that cell–cell contacts concomitantly induce both myosin II-driven contraction and protrusive branched actin-mediated extension^10^. Therefore, it would be reasonable to suppose that there is a mechanism to maintain contractility for persistent cell–cell junction shortening. In this study, we found that, in the absence of Pak3, aberrant actin dynamics at junctions disturb junction contraction during their shortening. Based on our results, we hypothesize that the following scenario occurs in cells lacking Pak3 (Fig. 5c): (i) upon contraction of cell–cell junctions, the actin dynamics at the junctions are altered to generate aberrant large protrusions that are rarely observed in control cells; (ii) since it is unlikely that actin protrusions will penetrate the cellular membrane, it is possible that the aberrant protrusions cause local unfolding of the membrane. Such unfolding causes a decrease in E-cadherin levels at the base of the protrusions on the cell–cell junctions; (iii) the decrease in junctional E-cadherin levels leads to the reduction of the connection sites between myosin II cables and AJs, which can weaken the interaction between myosin II cables and the AJs; and (iv) myosin II cables are not kept close to the junctions, which could cause insufficient transmission of their contractility to the junctions, leading to incomplete junction shortening. In normal conditions (in the presence of Pak3), Pak3 blocks the formation of such aberrant actin protrusions, maintains the E-cadherin levels at cell–cell junctions, places myosin II close to the junctions, and then ensures persistent junction contraction, which is necessary for the completion of cell–cell junction shortening (Fig. 5c). Since the E-Cad::GFP levels were not significantly decreased at the base of the small protrusions on the junctions, this scenario may hold true for only aberrant actin dynamics.

It should be noted that such aberrant actin protrusions may not be the only cause of the mislocalization of myosin II cables, since slight MRLC::GFP cable splitting was observed at the shortening junctions in the control cells at the late stage of junction shortening. Also, since Abi RNAi did not completely rescue the intercalation defect in Pak3 RNAi cells, it is possible that Pak3 regulates other proteins as well in this context (e.g., myosin II), and that branched actin structures are still—to some extent—required for A8a movement while the inhibition of these formations suppress the aberrant actin dynamics.

The scenario proposes a possible negative feedback mechanism (Fig. 5c, green arrows); the contractile forces of myosin II at junctions drive junction shortening, but they can oppositely perturb shortening by concomitantly altering actin dynamics, inducing the formation of WRC-dependent aberrant branched actin networks. Since Pak proteins are reported to regulate actin dynamics^54,55^, Pak3 is a candidate for counteracting such unnecessary actin dynamics, although its precise molecular mechanism is still unclear. Considering that Pak proteins and the WRC are activated by Rho GTPases^54,56,57^, it is possible that the presence of Pak3 sequesters these GTPases from the branching network-forming machineries. In addition, since Pak proteins are involved in a diverse array of biological events and have various substrates^24,26–31,58^, the identification of Pak3 substrates in this context will further deepen our understanding of how cells accomplish persistent junctional dynamics.

## Methods

### Fly genetics

The following *Drosophila melanogaster* stocks were used: *w*^*1118*^, *hs-flp*, *Act* > *CD2* > *Gal4*, *UAS-mCD8::mCherry*, *UAS-Lifeact::GFP*, *UAS-Lifeact::Ruby*, *His2Av::mRFP*, *UAS-Dicer2*, *UAS-MRLC* (*sqh*) *RNAi* (JF01103), *UAS-Abi RNAi* (HMC03190), *UAS-SCAR RNAi* (HMC03361), *UAS-β-catenin RNAi* (HMS01414), and *UAS-E-cadherin RNAi* (HMS00693) (Bloomington Drosophila Stock Center); *UAS-Pak3 RNAi* (39843), *UAS-Pak3 RNAi* (39844), *UAS-Pak3 RNAi* (107260), *UAS-MRLC RNAi* (7916) and *UAS-Rock RNAi* (3793) (Vienna Drosophila Resource Center); *UAS-Pak3 RNAi* (14895R-1) (National Institute of Genetics, Japan); *UAS-E-cadherin* (non-tagged full-length E-cadherin), *UAS-E-cadherin::GFP* (GFP-tagged full-length E-cadherin), and *Ubi-E-cadherin::GFP* (GFP-tagged full-length E-cadherin) (Drosophila Genomics and Genetic Resources); *UAS-Pak3::GFP* (a gift from S. Hayashi); *AbdB-Gal4*^*LDN*^ (ref. ^32^); *Pak3*^*d02472*^ (ref. ^40^); *E-Cad::GFP* (knock in, KI)^37^; *sqh*^*AX3*^*;sqh-sqh::GFP* (MRLC::GFP in the *MRLC*^*AX3*^ background)^46^; *sqh-UtrABD::GFP*^43^; and *UAS-Histone2B (H2B)::ECFP*^59^. Otherwise mentioned, the 14895R-1 strain was used to deplete Pak3. The flies were raised, and all experiments were performed at 25 °C except Supplementary Fig. 1 where the assay was performed at 29 °C. Somatic RNAi clones were induced using the FLP/FRT technique^60^ in white pupae (at 0 h APF) by heat shock (37 °C for 15 min). All the experiments were performed with male pupae during genitalia rotation (24 to 36 h after puparium formation) or within two days after adult eclosion, except the qPCR analysis where male wandering third instar larvae were subjected.

### Antibodies

Antibodies against Dlg (4F3; Developmental Studies Hybridoma Bank) and Pak3 (ref. ^28^) were used for primary antibodies. Alexa Fluor 647 goat anti-mouse IgG (A21236; Life Technologies) was used for a secondary antibody. All the antibodies were diluted at 1:200.

### Immunohistochemistry

Pupae were dissected and fixed in 4% paraformaldehyde in phosphate-buffered saline (PBS) for 20 min at room temperature (RT) and permeabilized with 0.1% Triton X-100 in PBS (PBT). The permeabilized samples were incubated in PBT with 5% donkey serum (blocking buffer) for 30 min at RT, incubated with primary antibodies in blocking buffer overnight at 4 °C, washed with PBT, incubated in blocking buffer for 30 min at RT, and incubated with secondary antibodies in blocking buffer for 2 h at RT. The samples were washed with PBT and mounted with 70% glycerol in PBS. Fluorescence microscopy images were captured on a TCS SP8 with a 63× numerical aperture (NA) 1.3 glycerol objective and LAS X (Leica). Images are maximum intensity projections of serial optical sections taken at a 0.5-μm z step size.

### Live imaging

Pupae were prepared as described previously^21^. In brief, the pupal case at the caudal region of male pupae was removed under a stereomicroscope, and the pupae were mounted on a glass slide with double-sided tape. A water-soaked filter paper and a small drop of water were placed around the pupae and on the caudal region, respectively, to avoid drying. The samples were covered with a cover glass, and the chamber was sealed with silicon. Time-lapse imaging of flies was performed using an SP8 confocal microscope with 63× NA 1.3 glycerol and 20× NA 0.75 dry objectives (Leica), except for the images in Figs. 1d, 3a, Supplementary Fig. 3, and Supplementary Movies 1, 2, which were obtained using an inverted microscope with a 60× NA 1.3 silicone oil objective (Olympus) equipped with a spinning-disc confocal unit (CSU-W1; Yokogawa) and a Zyla 4.2 PLUS sCMOS camera (Andor) on MetaMorph (Molecular Devises). All images are maximum intensity projections at the level of the AJs taken at a 1-μm z step size, except for images showing rotation of the genitalia (Fig. 1a), which are maximum intensity projections of the posterior end of flies taken at a 5-μm z step size. Time-lapse images were acquired at 10-s, 20-s, 2-min, or 10-min intervals.

### Tracking of junction dynamics

Junction dynamics and fluorescence intensities were analyzed manually with Fiji software. Projected time-lapse images were used. In the A8a epithelia, junctions were tracked from the initiation of movement (26 h APF) to the time when the genitalia angles were >90° (30 h APF)^21^. Junctions that were remodeled at least once were categorized as “remodeling.” Junctions that failed to resolve four-way vertices were categorized as “growth defect.” Junctions that remodeled, but then immediately retracted the new junctions and re-formed in the original direction were also categorized as “growth defect.” Junctions that shortened to <1.5 μm in length, but failed to form four-way vertices were categorized as “shortening defect.” “No remodeling” included junctions that did not shorten to <1.5 μm in length.

### Quantification of junction length, fluorescence intensity, and actin dynamics

Cell–cell junction length was determined as the distance between vertices. The junction lengths with split MRLC::GFP cables were determined as the distance between MRLC::GFP cables in cells surrounding the junctions when the MRLC::GFP cables in cells forming the junctions initiated splitting. The mean fluorescence intensity of E-Cad::GFP at a region along the junctions was measured using a line that covers the width of junctions. The junctional region at the base of protrusions was estimated in the presence of Lifeact::Ruby-positive protrusions. The intensity at the junctions in proximity to the bases was used for comparison with that at the bases. The frequency of the emergence of large actin protrusions was counted at each junction within 1 h.

#### Quantitative RT-PCR

Total RNAs were isolated from male wandering third instar larvae using Direct-zol RNA MiniPrep (R2050; ZYMO RESEARCH) and reverse-transcribed using PrimeScript RT reagent kit with gDNA Eraser (RR047A; TaKaRa). Quantitative PCR was performed using TB Green Premix Ex Taq II (RR820A; TaKaRa). Each mRNA level was normalized by *Rpl32* mRNA levels. Primers used were as follows: *E-cadherin*, 5′-CGGAGTGACGGGGCAC-3′ and 5′-TGGTTCACACCGCCGA-3′; *Rpl32*, 5′-CGGATCGATATGCTAAGCTGT-3′ and 5′-CGACGCACTCTGTTGTCG-3′.

### Statistical analysis

All statistical analyses were performed using R. To assess significance, the following tests were used: unpaired two-tailed *t*-test for comparing two groups; two-way analysis of variance (ANOVA) followed by Dunnett’s test for comparing the percentage of remodeling junctions and the sizes of actin protrusions between one group and the other groups (e.g., between the control and Pak3 mutants; between Pak3 RNAi and the rescue cells); two-way ANOVA followed by Tukey’s test for comparing junction length with split MRLC::GFP cables among more than two groups; one-sample *t*-test for the significance of the ratio (Int_base/_/Int_proximity_) with test values of 1 that represents no difference between these intensities; and unpaired two-tailed Mann–Whitney *U*-test followed by Bonferroni’s test for comparing the frequency of the appearance of the large actin protrusions.

### Reporting summary

Further information on research design is available in the Nature Research Reporting Summary linked to this article.

## Supplementary information

Supplementary Information

Description of Additional Supplementary Files

Supplementary Movie 1

Supplementary Movie 2

Supplementary Movie 3

Supplementary Movie 4

Supplementary Movie 5

Supplementary Movie 6

Reporting Summary

## Data Availability

Source data are provided as a Source data file. All the data included in the manuscript and Supplementary Information are also available from the corresponding author upon request. Source data are provided with this paper.

## Source data

Source Data
