# Supplementary material for: Inhibition of negative feedback for persistent epithelial cell–cell junction contraction by p21-activated kinase 3
Source: Nat Commun. 2022 Jun 20;13:3520. doi: 10.1038/s41467-022-31252-0 (PMC9209458; doi:10.1038/s41467-022-31252-0)
Supplement: Supplementary file 1 — Supplementary Information [file 41467_2022_31252_MOESM1_ESM.pdf]

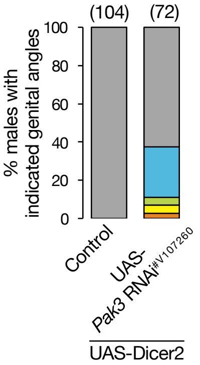

Supplementary Fig. 1 Percentages of male adult flies with genitalia angles indicated in Fig. 1b. Parentheses, the number of males examined. Genotypes: *+/Y;UAS-Dicer2/+;AbdB-Gal4, UAS-H2B::ECFP/+* and *+/Y;UAS-Dicer2/UAS-Pak3 RNAi (V107260);AbdB-Gal4, UAS-H2B::ECFP/+*. Source data are provided as a Source Data file.

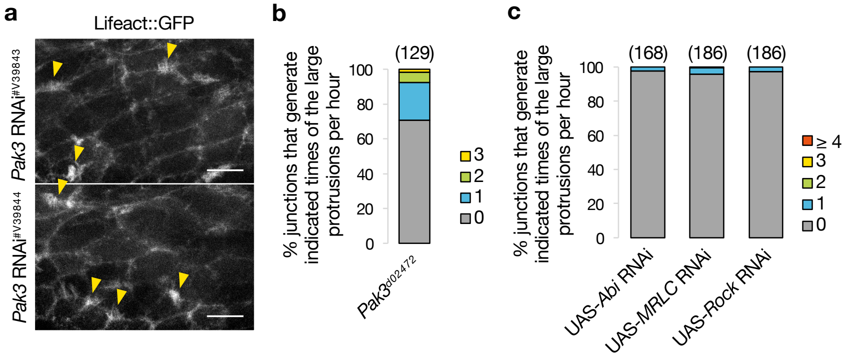

Supplementary Fig. 2 a Images of actin labeled with Lifeact::GFP in the A8a cells. Arrowheads indicate some aberrant protrusions. Scale bar, 10 μm. **b**, **c** Percentages of junctions forming the large actin protrusions for the indicated number of times per hour are shown. Parentheses, the number of examined junctions from 2–4 pupae per genotype. In **b** the large actin protrusions were defined with UtrABD::GFP signals instead. Genotypes: **a** *+/Y;UAS-Lifeact::GFP/+;AbdB-Gal4/UAS-Pak3 RNAi (V39843 or V39844)*; **b** *+/Y;sqh-UtrABD::GFP/+;Pak3*^{d02472}; **c** *+/Y;UAS-Lifeact::GFP/UAS-Abi RNAi;AbdB-Gal4/+*, *+/Y;UAS-Lifeact::GFP/UAS-MRLC RNAi;AbdB-Gal4/+*, and *+/Y;UAS-Lifeact::GFP/UAS-Rock RNAi;AbdB-Gal4/+*. Source data are provided as a Source Data file.

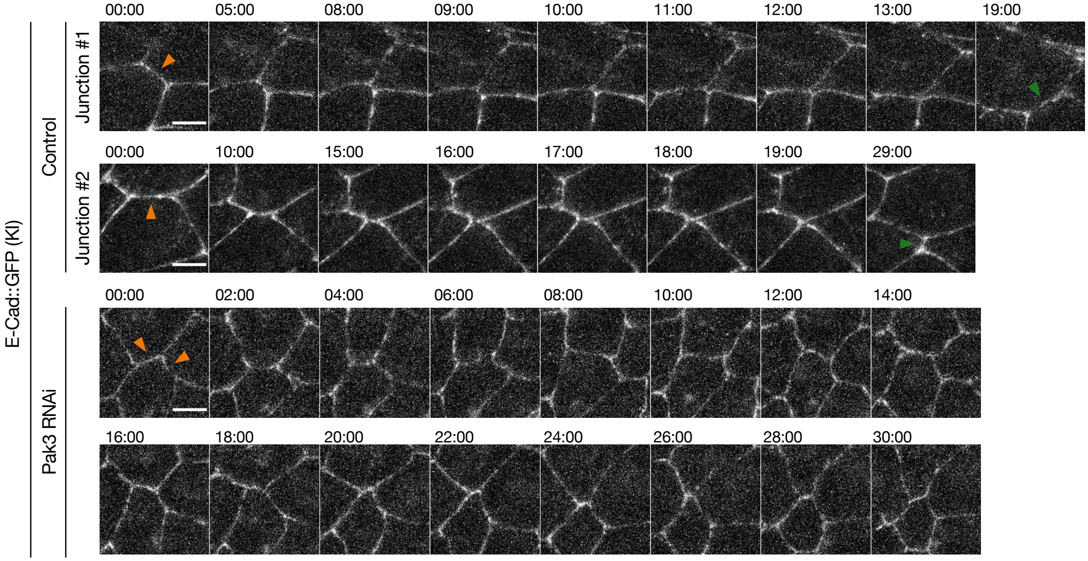

Supplementary Fig. 3 Additional representative time-lapse images of E-Cad::GFP at remodeling (control) and shortening (Pak3 RNAi) junctions, which are indicated with orange arrowheads, from >3 biologically independent pupae. Scale bar, 5 μm. Genotype: *+/Y;E-Cad::GFP (KI)* and *+/Y;E-Cad::GFP (KI);AbdB-Gal4, UAS-H2B::ECFP/UAS-Pak3 RNAi*.

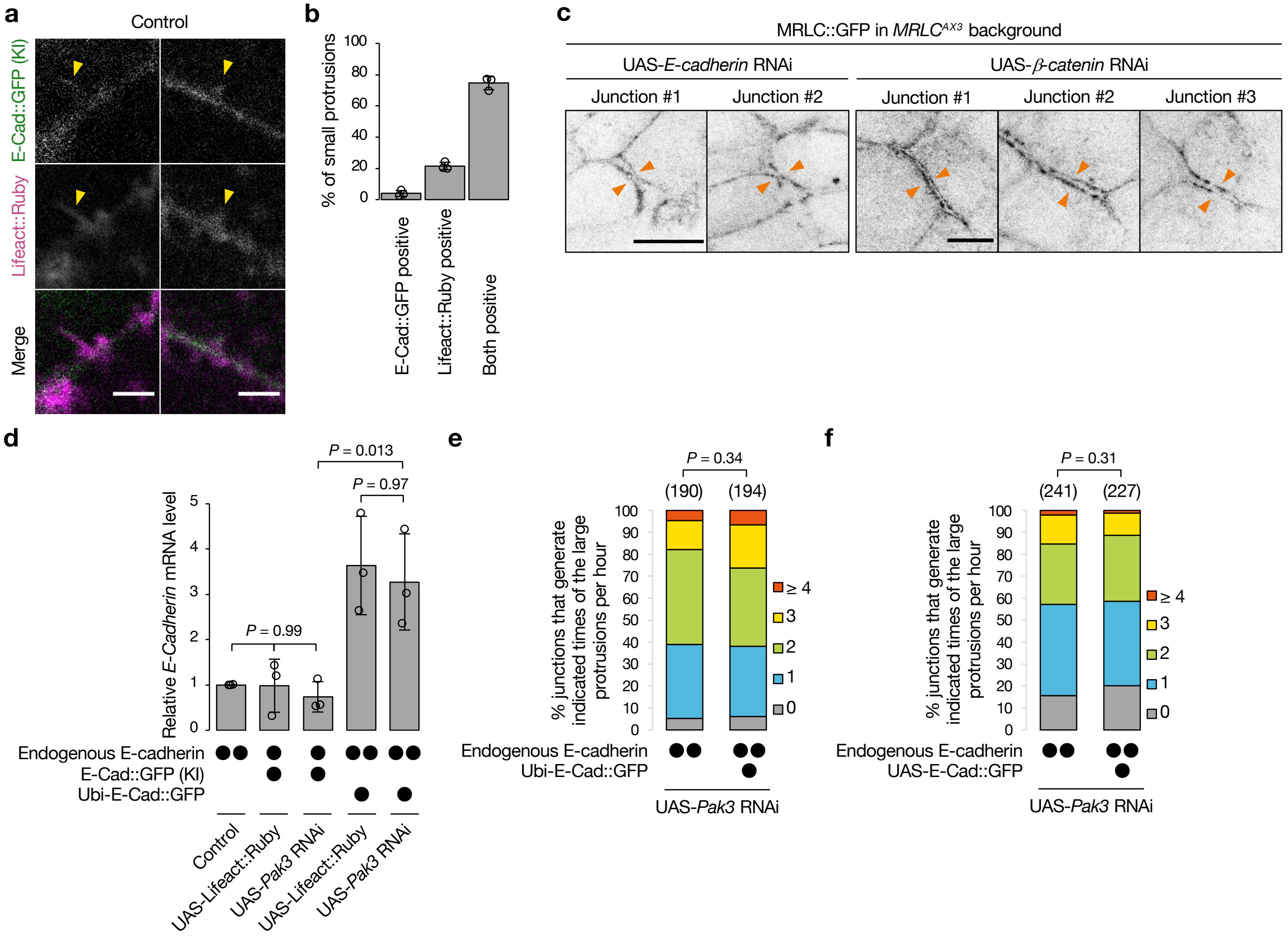

Supplementary Fig. 4 a Images of junctions with E-Cad::GFP and Lifeact::Ruby in the control cells. Arrowheads indicate E-Cad::GFP and Lifeact::Ruby double-positive protrusions. Scale bar, 2 µm. **b** The mean ± S.D. of the percentages of small protrusions positive for the indicated signals in the control cells. 221 protrusions from *n* = 3 biologically independent pupae were examined. **c** Representative images of split MRLC::GFP cables at junctions in the AJ components-depleted cells. Scale bar, 10 µm. **d** Mean ± S.D. of relative *E-cadherin* mRNA levels normalized to that of *Rpl32*. *n* = 3 independent experiments. Dots indicate the copy number of each E-cadherin allele. P-values by two-tailed Tukey's test. **e, f** Percentages of junctions forming the large actin protrusions for the indicated number of times per hour are shown. Parentheses, the number of examined junctions from 4 pupae per genotype. Dots indicate the copy number of each E-cadherin allele. *P*-values by the two-tailed Mann-Whitney *U*-test. Genotypes: **a, b** *+/Y;E-Cad::GFP (KI)/UAS-LifeAct::Ruby;AbdB-Gal4/+*; **c** *MRLC^{AX3}/Y;MRLC-MRLC::GFP/+ RNAi;AbdB-Gal4, UAS-H2B::ECFP/UAS-E-cadherin RNAi* and *MRLC^{AX3}/Y;MRLC-MRLC::GFP/+ RNAi;AbdB-Gal4, UAS-H2B::ECFP/UAS-β-catenin RNAi*; **d** *+/Y* (Control), *+/Y;E-Cad::GFP (KI)/UAS-Lifeact::Ruby;AbdB-Gal4/+*, *+/Y;E-Cad::GFP (KI)/UAS-Lifeact::Ruby;AbdB-Gal4/UAS-Pak3 RNAi*, *+/Y;Ubi-E-Cad::GFP/UAS-Lifeact::Ruby;AbdB-Gal4/+*, and *+/Y;Ubi-E-Cad::GFP/UAS-Lifeact::Ruby;AbdB-Gal4/UAS-Pak3 RNAi*. **e** *+/Y;UAS-Lifeact::GFP/+;AbdB-Gal4/UAS-Pak3 RNAi* and *+/Y; UAS-Lifeact::GFP/Ubi-E-Cad::GFP;AbdB-Gal4/UAS-Pak3 RNAi*; **f** *+/Y; UAS-Lifeact::GFP/+;AbdB-Gal4, UAS-H2B::ECFP/UAS-Pak3 RNAi* and *+/Y; UAS-Lifeact::GFP/UAS-E-Cad::GFP;AbdB-Gal4/UAS-Pak3 RNAi*. Source data are provided as a Source Data file.
