# Supplementary material for: Inhibition of negative feedback for persistent epithelial cell–cell junction contraction by p21-activated kinase 3
Source: Nat Commun. 2022 Jun 20;13:3520. doi: 10.1038/s41467-022-31252-0 (PMC9209458; doi:10.1038/s41467-022-31252-0)
Supplement: Supplementary file 2 — Description of Additional Supplementary Files [file 41467_2022_31252_MOESM2_ESM.pdf]

Description of Additional Supplementary Files

Supplementary Movie 1. Dynamics of cell–cell junctions during the movement of control A8a cells. E-cadherin was visualized with E-Cad::GFP. Scale bar, 10 μm. Genotype, *+/Y;E-Cad::GFP (KI);AbdB-Gal4, UAS-H2B::ECFP/+*.

Supplementary Movie 2. Dynamics of cell–cell junctions during the movement of Pak3 RNAi A8a cells. E-cadherin was visualized with E-Cad::GFP. Scale bar, 10 μm. Genotype*, +/Y;E-Cad::GFP (KI);AbdB-Gal4, UAS-H2B::ECFP/UAS-Pak3 RNAi*.

Supplementary Movie 3. Dynamics of actin at cell–cell junctions in the control A8a cells. Actin was visualized with Lifeact::GFP. Scale bar, 10 μm. Genotype, *+/Y;UAS-Lifeact::GFP;AbdB-Gal4/+*.

Supplementary Movie 4. Dynamics of actin at cell–cell junctions in the Pak3 RNAi A8a cells. Actin was visualized with Lifeact::GFP. Scale bar, 10 μm. Genotype, *+/Y;UAS-Lifeact::GFP;AbdB-Gal4/UAS-Pak3 RNAi*.

Supplementary Movie 5. Dynamics of E-Cad::GFP at cell–cell junctions in the control A8a cells. Scale bar, 3 μm. Genotype, *+/Y;E-Cad::GFP (KI);AbdB-Gal4, UAS-H2B::ECFP/+*.

Supplementary Movie 6. Dynamics of E-Cad::GFP at cell–cell junctions in Pak3 RNAi A8a cells. Scale bar, 3 μm. Genotype, *+/Y;E-Cad::GFP (KI);AbdB-Gal4, UAS-H2B::ECFP/UAS-Pak3 RNAi*.
