# Supplementary material for: Inhibition of negative feedback for persistent epithelial cell–cell junction contraction by p21-activated kinase 3
Source: Nat Commun. 2022 Jun 20;13:3520. doi: 10.1038/s41467-022-31252-0 (PMC9209458; doi:10.1038/s41467-022-31252-0)
Supplement: Supplementary file 9 — Reporting Summary [file 41467_2022_31252_MOESM9_ESM.pdf]

nature portfolio

Corresponding author(s): Erina Kuranaga

Last updated by author(s): Jun 3, 2022

Reporting Summary

Nature Portfolio wishes to improve the reproducibility of the work that we publish. This form provides structure for consistency and transparency in reporting. For further information on Nature Portfolio policies, see our Editorial Policies and the Editorial Policy Checklist.

Statistics

For all statistical analyses, confirm that the following items are present in the figure legend, table legend, main text, or Methods section.

n/a	Confirmed	
☐	☒	The exact sample size (*n*) for each experimental group/condition, given as a discrete number and unit of measurement
☐	☒	A statement on whether measurements were taken from distinct samples or whether the same sample was measured repeatedly
☐	☒	The statistical test(s) used AND whether they are one- or two-sided<br>*Only common tests should be described solely by name; describe more complex techniques in the Methods section.*
☒	☐	A description of all covariates tested
☐	☒	A description of any assumptions or corrections, such as tests of normality and adjustment for multiple comparisons
☐	☒	A full description of the statistical parameters including central tendency (e.g. means) or other basic estimates (e.g. regression coefficient) AND variation (e.g. standard deviation) or associated estimates of uncertainty (e.g. confidence intervals)
☐	☒	For null hypothesis testing, the test statistic (e.g. *F*, *t*, *r*) with confidence intervals, effect sizes, degrees of freedom and *P* value noted<br>*Give P values as exact values whenever suitable.*
☒	☐	For Bayesian analysis, information on the choice of priors and Markov chain Monte Carlo settings
☒	☐	For hierarchical and complex designs, identification of the appropriate level for tests and full reporting of outcomes
☒	☐	Estimates of effect sizes (e.g. Cohen's *d*, Pearson's *r*), indicating how they were calculated

Our web collection on statistics for biologists contains articles on many of the points above.

Software and code

Policy information about availability of computer code

Data collection	Leica LAS X (version 2.0.1) and Metamorph (version 7.7.5.) for image acquisition.
Data analysis	Fiji (ImageJ, version 2.3.0) for image analyses; R (version 4.0.3) for statistical analyses.

For manuscripts utilizing custom algorithms or software that are central to the research but not yet described in published literature, software must be made available to editors and reviewers. We strongly encourage code deposition in a community repository (e.g. GitHub). See the Nature Portfolio guidelines for submitting code & software for further information.

Data

Policy information about availability of data

All manuscripts must include a data availability statement. This statement should provide the following information, where applicable:

- Accession codes, unique identifiers, or web links for publicly available datasets
- A description of any restrictions on data availability
- For clinical datasets or third party data, please ensure that the statement adheres to our policy

All the data are included in the manuscript, Supplementary Information, and a Source Data. They are also available from the corresponding author upon request.

Field-specific reporting

Life sciences study design

All studies must disclose on these points even when the disclosure is negative.

Sample size	No sample-size calculation was performed. Sample sizes were chosen based on our previous studies (Sato et al, Nat Commun, 2015; Uechi and Kuranaga, Dev cell, 2019), and those are similar to other studies in this research field (Coutelis et al, Dev Cell, 2013; Collinet et al, Net Cell Biol, 2015).
Data exclusions	Image data were excluded if the cells showed quite weak signals due to unhealthy samples or failure in sample preparation.
Replication	At least two animals were examined to collect data points and to ensure the reproducibility in each experiment.
Randomization	Samples were grouped by genotype.
Blinding	No blinding was performed since it is impossible to retrieve the samples mounted on the slide glasses after live imaging, and the genotype information for grouping cannot be determined.

Reporting for specific materials, systems and methods

We require information from authors about some types of materials, experimental systems and methods used in many studies. Here, indicate whether each material, system or method listed is relevant to your study. If you are not sure if a list item applies to your research, read the appropriate section before selecting a response.

Materials & experimental systems

n/a	Involved in the study	
☐	☒	Antibodies
☒	☐	Eukaryotic cell lines
☒	☐	Palaeontology and archaeology
☐	☒	Animals and other organisms
☒	☐	Human research participants
☒	☐	Clinical data
☒	☐	Dual use research of concern

Methods

n/a	Involved in the study	
☒	☐	ChIP-seq
☒	☐	Flow cytometry
☒	☐	MRI-based neuroimaging

Antibodies

Antibodies used	Antibodies used are described in the method section: anti-Dlg (4F3, Developmental Studies Hybridoma Bank); anti-Pak3 (a gift from Dr. N. Harden, Development, 2010, doi:10.1242/dev.045088); Alexa Fluor 647 goat anti-mouse IgG (A32728, Life Technologies). All the antibodies were used at 1:200 dilution.
Validation	Anti-Dlg antibody<br>It is widely used, and the signal pattern was consistent to that in the previous studies. Its web site is: https://dshb.biology.uiowa.edu/4F3-anti-discs-large<br><br>Anti-Pak3 antibody<br>Generation of this antibody was described in a previous paper (Bahri, S. et al. The leading edge during dorsal closure as a model for epithelial plasticity: Pak is required for recruitment of the Scribble complex and septate junction formation. Development (2010). doi:10.1242/dev.045088). Signal specificity was confirmed by Pak3 RNAi.

Animals and other organisms

Policy information about studies involving animals; ARRIVE guidelines recommended for reporting animal research

Laboratory animals	Drosophila melanogaster was used. Male pupae during metamorphosis in the time window from 24 to 36 h after puparium formation were imaged for molecular and cellular dynamics. Adult male flies were examined for resultant tissue (genital) dynamics within two days after eclosion. For qPCR, male wandering third instar larvae were used.
Wild animals	This study did not involve wild animals.
Field-collected samples	This study did not contain field-collected samples.
Ethics oversight	No ethical approval was required.

Note that full information on the approval of the study protocol must also be provided in the manuscript.
